# Surface Properties of Graphene Functionalized TiO_2_/nHA Hybrid Coatings Made on Ti6Al7Nb Alloys via Plasma Electrolytic Oxidation (PEO)

**DOI:** 10.3390/molecules26133903

**Published:** 2021-06-25

**Authors:** Oktay Yigit, Niyazi Ozdemir, Burak Dikici, Mosab Kaseem

**Affiliations:** 1 Department of Metallurgical and Materials Engineering, Firat University, Elazig 23119, Turkey; oyigit@firat.edu.tr (O.Y.); nozdemir@firat.edu.tr (N.O.); 2 Department of Metallurgical and Materials Engineering, Ataturk University, Erzurum 25240, Turkey; 3 Department of Nanotechnology and Advanced Materials Engineering, Sejong University, Seoul 05006, Korea; mosabkaseem@sejong.ac.kr

**Keywords:** graphene, PEO, Ti6Al7Nb, hydroxyapatite, in-vitro corrosion

## Abstract

Nano-hydroxyapatite (nHA)-matrix coatings containing graphene nanosheets (GNS)-nHA were coated on Ti6Al7Nb alloys by plasma electrolytic oxidation (PEO) treatment for the improvement of their surface properties. Crystallographic properties, functional groups, and elemental analysis of coatings were characterized by XRD, ATR–FTIR, and EDS analysis. Surface morphological changes of the coated surfaces were investigated by AFM and SEM. The electrochemical corrosion behavior of the coatings was examined by using the potentiodynamic scanning (PDS) tests under in-vitro conditions in simulated body fluid (SBF). The results showed that the GNS was successfully deposited in ceramic matrix coatings on Ti6Al7Nb alloys. Also, the microstructural observations revealed that the coatings have a porous and rough structure. The XRD and ATR–FTIR quantitative analysis have proved the appearance of HA and GNS in the coating layers. An increase in the coating thickness, surface hardness, and anatase/rutile transformation rate was determined, while the GNS ratio in the coating layers was increased. The microhardness of the nHA coating reinforced with 1.5 wt% GNS was measured at 862 HV, which was significantly higher than that of GNS-free (only nHA) coating (584 HV). The best in-vitro resistance to corrosion in SBF was observed in the nHA/1.5GNS wt% coating.

## 1. Introduction

Due to the development of technology, the use of new technologies in medical science has also increased, and with this integration, new developments and applications have brought many innovations in implant production. New generation biomaterials give strength and courage to repair and exchange functional tissues in the body. It is well known that biomaterials must meet the vital, mechanical, and physical demands of their users. Titanium (Ti) and its alloys are preferred material groups as implant materials in this industry branch [1]. Despite low cell adhesion capabilities with bone, Ti alloys are preferred due to their high strength, good corrosion resistance, and good biocompatibility properties in many orthopedic applications [2,3].

In recent years, Ti6Al7Nb alloys have been proposed instead of commercially Ti6Al4V alloys due to the ion release of Vanadium (V) in particular. Moreover, new tissue formation ability (osseointegration) after biological implantation of the Ti6Al4V alloys at the bone/implant interfaces is relatively lower than that in Ti6Al7Nb alloys. By the way, the osseointegration depends on the chemical composition, topographic properties, morphology, and corrosion resistance of the implant surface. Thus, it is necessary to use modification techniques such as surface coatings or functionalization to improve the biocompatibility of the implant candidate.

Different surface modification techniques and hybrid compositions are used in titanium implants to increase their surface bioactivity. These techniques include the sol-gel method [4,5,6], plasma spraying method [7], ion implantation [8], laser surface modification [9], electrophoretic deposition [10], anodic oxidation [11], ultrasonic method [12], biomimetic mineralization process [13], spark plasma sintering (SPS) [14], cold vacuum spray [15], hydrothermal method [16], and plasma electrolytic oxidation (PEO) [3,17,18]. The PEO is known as micro-arc oxidation and is used to produce ceramic coatings with various thicknesses and higher hardness on generally light metals and their alloys.

The coatings produced by the PEO method have high adhesion and abrasion resistance. In addition, highly porous coatings with good corrosion protection properties can be obtained easily without additional treatment. The importance of the porous structure facilitates cell attachment on the implant surfaces. Another advantage of the PEO method has been completely covering implant surfaces with bioactive components. The protective oxide layer prevents body fluid corrosion and does not cause allergies, clot formation, and inflammation due to any reaction in the tissues [18]. This stable oxide layer acts also as a barrier to the release of ions from the substrate to the body tissue and solves the problems associated with ion accumulation during the extended time usage of the implant.

In several studies, the formation mechanisms [19] and bioactivity behaviors [20] of PEO coatings on the surface of Ti alloys have been investigated by using different process parameters and electrolyte baths that have different chemical components. Current (A), Voltage (V), frequency (f), duty time (DS), operating mode, and electrolyte properties used in the PEO process determine the quality of PEO coatings [21]. The properties of the PEO coatings produced on the alloy surfaces especially depend on the substrate and electrolyte composition elements. In particular, calcium (Ca) and phosphate (P) elements from the electrolyte are embedded into oxide coatings for obtaining bioactivity during the PEO process. Synthetic apatite-based bioactive ceramics such as hydroxyapatite (HA), tri-calcium phosphate (TCP), biphasic calcium phosphate (BCP), or their derivatives were doped in the coatings [18,22].

The corrosion behavior of oxidized ceramic coatings in simulated body fluid (SBF) has been investigated and it has been reported that electrolyte properties affect corrosion resistance [18]. According to literature outputs, the corrosion resistance of the PEO coatings increases with increasing coating thickness and reduced pore diameters [23]. A low corrosion rate and high mechanical strength are crucial to the long-term success of the implant.

In recent years, hydroxyapatite and a graphene hybrid structure have been coated during the PEO treatment on Mg alloys [24,25]. However, to the best of our knowledge, the coating of the hybrid structure on the Ti6Al7Nb by PEO has not been reported until now.

Zou et al. [26] studied the effect of graphene oxide (GO) concentration on the tribo-corrosion behavior of the PEO coatings on Ti6Al4V alloys. The corrosion susceptibilities of the coatings were analyzed by using electrochemical measurements and it was found that the addition of GO enhanced the tribocorrosion resistance of the PEO coated Ti samples. In another study, Li et al. [27] prepared a two-stage composite in the Ti6Al4V alloy with a PEO and sol-gel coated layers with graphene oxide doping and then examined the corrosion resistance in seawater. They reported that the micropores in the PEO film were filled with the GO/sol-gel layer and the formation improves the corrosion performance of the substrates through the suitable barrier property of the GO nanosheets. Liu et al. [28] investigated the effects of GNS on ceramic coatings deposited on the Ti6Al4V via the PEO method. They found that the GNS layers exhibited a physical barrier and significantly increased wear resistance and hardness of the coatings.

The above studies showed that surface properties, biocompatibility, and corrosion resistance of the PEO coatings are essential points to consider for an implant candidate. Thus, a systematic study was done on the nHA coatings reinforced with GNS on the Ti6Al7Nb alloys in this study. The coatings’ morphological changes and in-vitro corrosion resistance were investigated and compared with their unreinforced counterparts (only HA coatings). The effect of the GNS additive on the hydroxyapatite nucleation was discussed in detail. This study aims to enhance the biological, mechanical, and chemical properties of Ti6Al7Nb alloy surfaces by PEO treatment using the nHA and GNS combined structure.

## 2. Results and Discussion

**Figure 1:**
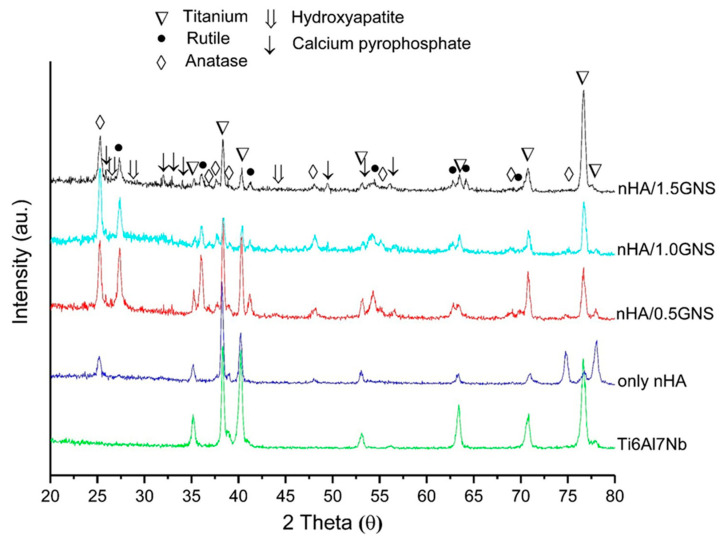
XRD patterns of the nHA/GNS coated and uncoated Ti6Al7Nb samples.

XRD graphics of the nHA/GNS coated and uncoated Ti6Al7Nb samples are given in Figure 1 comparatively. Anatase and rutile oxides, hydroxyapatite with very low intensities, and calcium pyrophosphate were mainly determined in the coating layers. Also, titanium phases from the substrates appeared in the XRD graphs. However, the prominent peaks of hydroxyapatite were not pronounced, and a decrease in the peak of the phase was observed with GNS addition. This reduction in peak intensity is due to the nucleation of HA crystals on the oxidized surfaces during the PEO. It was possible that this decrease can be explained by the nucleation and growth mechanism of HA crystals on graphene surface. That means more nano-sized HAs can be seen on the surface but most of them show an amorphous structure [29].

The two main phases of the TiO_2_ (rutile and anatase), which are expected to occur after oxidation due to the nature of the PEO process, were observed in the XRD peaks. However, the peaks containing Ca- and P- were observed at very low intensities. Similar observations have been reported by Yerokhin et al. [3] and Wang et al. [30]. The researchers have implied that there are Ca and P elements in the structure; although they are not seen in the graphics, they may be in an amorphous form in the TiO_2_ phase. In addition, dissolved Ca- and P- elements in the titanium oxide matrix without the formation of any significant crystalline phase were observed by Fazel et al. [31] and the researchers reported that even though no Ca- and P- crystalline structures were determined in the XRD analysis of PEO layers, the presence of Ca- and P- elements was proved by EDS in the coating layers. The reason for the narrow and low intensities is due to the nano-sized HA crystals.

In this study, the rutile (02-0494) and anatase (01-0562) phases were obtained in all experimental conditions, although the peak values were slightly different. The peak of the rutile TiO_2_ phase was not seen in the PEO coatings without GNS additives. In other words, although there was a significant change in the anatase phase with an increasing GNS additive, the rutile phase peaks appeared with the GNS additive. It can be said that the peaks rutile phase peaks increased significantly with the GNS additive. The anatase phase occurs earlier than the rutile phase because the temperature is lower in the micro discharge channels at low coating times compared to the higher coating times. At the same time, the rutile phase is thermodynamically more stable than the anatase phase at higher temperatures. The conversion from anatase to rutile phase in the TiO_2_ structure occurs at about 680 °C (953K). It can be thought that the GNS addition during the PEO treatment acted as a barrier to the forming of the coating and caused the temperature to remain above 680 °C, thereby forming a more stable rutile TiO_2_ phase instead of the anatase TiO_2_ occurring at lower temperatures on the surfaces. Meanwhile, the solubility of Ti metal ions in the thermodynamically more stable rutile phase is lower than the metastable anatase phase, so rutile TiO_2_ is preferred over the anatase TiO_2_ phase for biomedical purposes [32]. Both anatase and rutile phases are well-known to have superior anti-bacterial, bioactive, and biocompatible structures for bone implantation in biomedical fields [33]. TiO_2_ phases such as anatase and rutile phases adhere to hydroxyapatite chemically due to their high bioactivity, increasing the adhesion strength between hydroxyapatite and titanium [34]. Lastly, main Ti peaks (44-1288) were observed at the coatings. Also, a form of Ca-P phase, calcium pyrophosphate (09-0345) peaks were observed.

**Figure 2:**
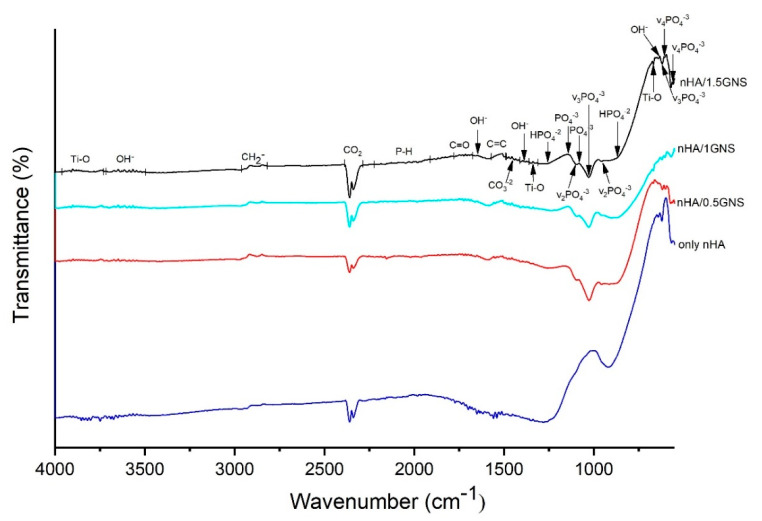
ATR–IR analyzed results of the nHA/GNS coated samples with different GNS additive ratios on Ti6Al7Nb alloy.

The ATR–IR analysis results of the only nHA and nHA/GNS coated Ti6Al7Nb samples are presented in Figure 2. According to the literature, the first sign of the presence of HA in the composite coatings is the wide ATR–IR bands between the range of 960–1135 cm^−1^. Also, OH^−^ peaks around 3500–3700 cm^−1^ are characteristic peaks of stoichiometric hydroxyapatite [35,36].

The wavelengths of 632–635, 1645, and 3503–3725 cm^−1^ were determined as ν_L_ and ν_s_ (OH^−^) bands and hydroxyapatite phase, respectively. The existence of hydroxyapatite and different apatite phases were verified with ν_3_ (PO_4_^−3^) and bands in the numbers of 1912–2225, 1027, and 620 cm^−1^ wavenumbers. The bands between 2292–2391 cm^−1^ corresponded to typical CO_2_ and usually come from atmospheric carbon dioxide, which is inevitably absorbed by water during the coating process. Apatite structure was determined with ν_1_ (HPO_4_^−2^) bands in 867–872 and 1255 cm^−1^ wave numbers. Hydroxyapatite and apatite phases were determined in 960, 1105, and 1081–1135 cm^−1^ wavenumbers with ν_2_ (PO_4_^−3^) asymmetric stretching, and ν_1_ (PO_4_^−3^) asymmetric turning bands, respectively. The presence of carbonate groups (CO_3_^−2^) in the of 1414–1488 cm^−1^ could be seen. The location of the carbonate bands proves the presence of predominantly β type HA, the preferred substitute in human bones, known for its excellent bioactivity and osteoinductivity [37,38]. The anatase phase of TiO_2_ was detected by the ν (Ti–O) band between the 3730–3920 cm^−1^ wavenumbers. The TiO_2_ phase structure was determined with δ (Ti–O) and v (Ti–O) bands in 671 and 1312–1360 cm^−1^ wavenumbers, respectively. The most significant change was the occurrence of the C=C band acquired in the wave range of 1490–1570 cm^−1^ in comparison to the additive-free samples with the increasing GNS addition in the coating layer (Figure 2). It has been reported that this band proves GNS’s presence and corresponds to the skeletal vibration of graphene due to sp^2^ hybridized C=C vibration stress [29,37,39,40,41,42,43]. The presence of GNS in the PEO coated samples was confirmed with the occurrence of this band.

Peak intensity changes in the phosphate group also occurred with the increase of the GNS addition to the coating layer. This decrease in peak intensity is quite evident, especially in 0.5 wt% and 1.0 wt% GNS reinforcement rates. However, an increase in peak intensity was observed in the 1.5 wt% GNS reinforcement rate. Therefore, due to the increase in GNS additive, it can be thought that HA crystals can find more areas where they can attach to the GNS surfaces due to the increased surface area. It can be also considered that the number of phosphate groups converted into the hydroxyapatite crystals at 0.5 wt% and 1.0 wt% GNS additive rates is higher than 1.5 wt% GNS additive coatings.

HA crystals were also detected in 1.5 wt% GNS additive coatings. XRD graphics and ATR-IR analysis confirms that HA structures exhibit nano-sized structures compared to coated samples with lower GNS reinforcement ratios. Larger peaks in the ATR-IR spectrum prove the presence of nanostructures in the coating structure [44]. It can be said that NHA/GNS composites are effectively embedded in surface coatings on Ti6Al7Nb substrates. Findings obtained as a result of the study are compatible with different literature outputs. Although a literature comparison has been made, this study is the first study in which nHA/GNS hybrid coatings were synthesized by the PEO method using Ti6Al7Nb alloy. In our previous study, Ti6Al4V alloy was coated and analyzed similarly [45]. However, some differences were observed in these studies using a different kind of Ti alloy. Therefore, comparisons of refs. [24,35,36,37,40,46,47,48,49,50] have been identified by a mixed review of GNS-doped hydroxyapatite coatings and surface coatings made with PEO.

**Figure 3:**
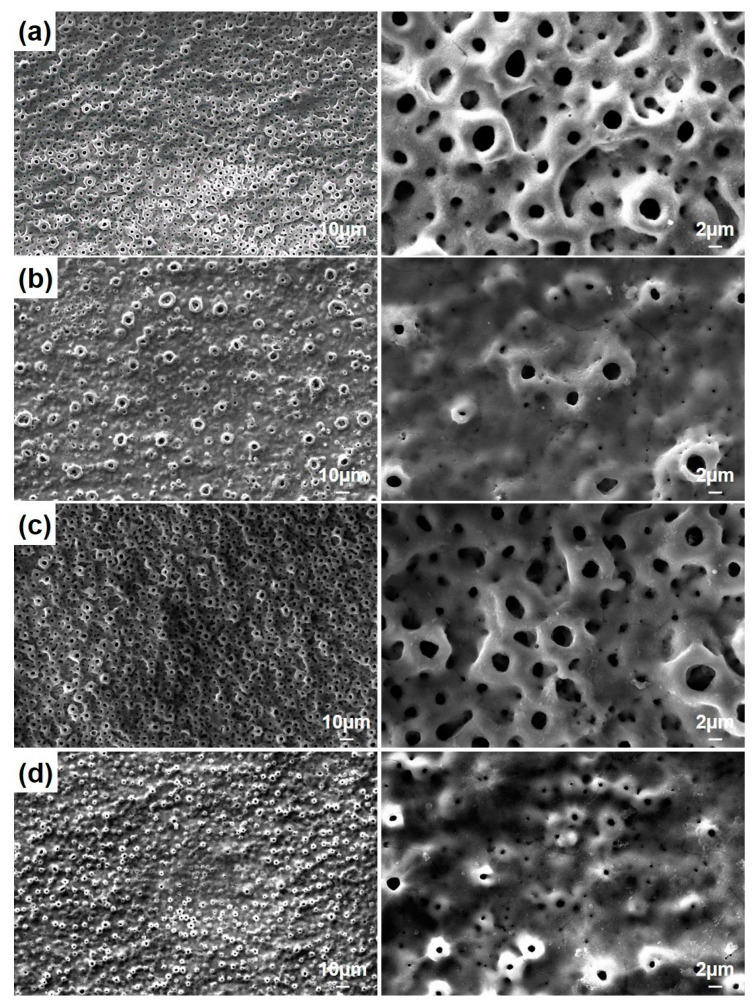
The SEM morphologies of PEO treated coatings with (**a**) free GNS, (**b**) 0.5, (**c**) 1.0, and (**d**) 1.5 wt% GNS additive at different magnifications.

Figure 3 shows the surface morphologies of the nHA/GNS hybrid coatings after PEO treatment. All coatings had a typical porous oxide structure reported by other researchers [3,31]. It was seen that the pore diameters are significantly reduced in nHA coating reinforced with 0.5 wt% GNS additive. It was also observed that the pore diameters of the coating decreased with the addition of GNS while the number of pores increased. This was due to the metal thrown out from the micro discharge channels formed by sparks because the GNS blocks the channels. It can be said that the GNS additive prevents the growth of micro-sparking pores [28]. However, a small increase in the pore size was determined in the coating reinforced with 1.0 wt% GNS (Figure 3c). In other words, the surface morphology of the coating was somehow similar to the case of only nHA coating (free–GNS coating). It is worth mentioning that the pore size of coatings was affected not only by the GNS deposition ratio during the PEO treatment but also by electrolyte properties. In 0.5 wt% GNS additive coatings, the conductivity of the electrolyte was measured as 14.02 mS·cm^−1^ and 1.0 wt% GNS coating was 15.02 mS·cm^−1^. That means lower GNS additive rates still blocked the micro discharge channels. But with an increase in the GNS ratio, the conductivity of electrolyte also increased significantly and the pore size increased after PEO treatment. In that case, the GNS additive with a 1.0 wt% rate can change the intensity of the micro sparks. Therefore, it is possible to mention a certain critical rate for GNS addition. Nevertheless, the pore diameters again decreased significantly with the addition of 1.5 wt% GNS. It could be concluded that the number of pores increases with increasing GNS additions.

**Figure 4:**
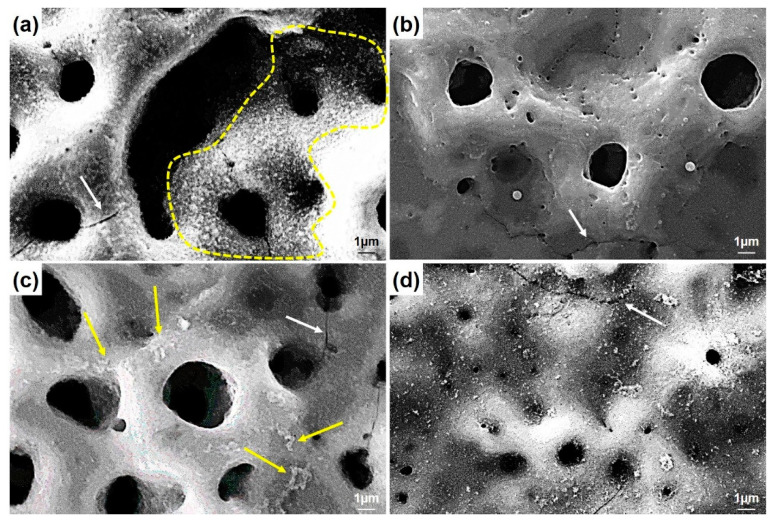
The SEM morphologies of PEO treated coatings with (**a**) free GNS, (**b**) 0.5, (**c**) 1.0, and (**d**) 1.5 wt% GNS additive at higher magnifications.

**Figure 5:**
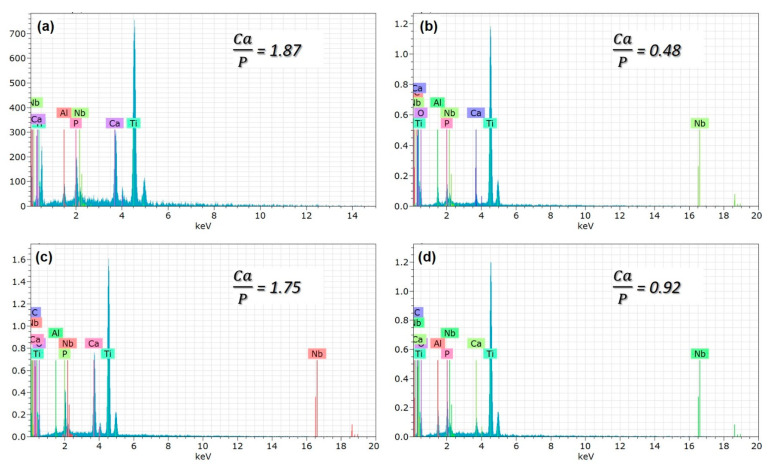
The EDS analysis of PEO treated coatings with (**a**) free GNS, (**b**) 0.5, (**c**) 1.0, and (**d**) 1.5 wt% GNS additive.

The higher magnifications of the surfaces are presented in Figure 4. The nHA nucleation was observed on the GNS-free coating (the only HA-containing coating) surfaces with the nano-size (dashed-line area in Figure 4a). The HA crystals accumulated in the inner part of the pores can be seen easily in the images. Some clustered structures were also observed on the GNS containing coating surfaces (marked with arrows in Figure 4c). The EDS results confirmed that these crystallized clusters were Ca- and P-based derivatives such as HA (Figure 5). The hybrid structure of the nHA and GNS containing clusters, which was probably due to the higher GNS ratio in the sample, was also revealed fully in 1.5 wt% containing the GNS coating surface (Figure 4d). The transformation of the hydroxyapatite structure from the amorphous phase to more crystalline nano phases increased, and this transformation became most pronounced in the nHA/1.5GNS coating. Therefore, it is thought that HA crystals can attach better to GNS layers and adhere to these surfaces due to the increase in the GNS additive ratio. Wen et al. [24] stated that the HA (300) plane could naturally bond to the graphene surface, creating a solid and consistent interface bond thanks to the Van der Waals bond. Due to this feature, they stated that graphene might help to hold HA particles in a PEO coating. Also, the GNS additive caused the coating temperature to cool slowly during coating, resulting in higher crystallinity structures rather than amorphous hydroxyapatite structures. According to Figure 4, microcracks (indicated with white arrows) proceeded directly through the pores, not through the interfaces of the pores in the coatings reinforced with 1 wt% GNS, probably because larger-size pores may be caused by stress accumulation in the coating layer.

The thickness of coatings, porosity features, and surface areas of coated Ti6Al7Nb alloys with nHA/GNS by the PEO method are given in Table 1.

According to Table 1, a decrease in coating thickness and the porosity diameter occurred when 0.5 wt% GNS was added compared to free–GNS coatings. However, a significant increase in the parameters of the 1.0 wt% GNS containing coating was determined. In this case, increasing the porosity size caused a significant increase in the coating thickness (Figure 3 and Table 1). Besides, the GNS additive provided significant differences in the number of pores (Table 1). In the sample reinforced with 1.5 wt% GNS, the decrease in its diameter with an increasing pore number was remarkable. It was also seen that nHA and graphene covered some tiny pores in the coatings. A lower pore rate may have occurred in PEO treated coatings due to HA and graphene-impermeable partial pores due to the electric field during the PEO process [24,28]. Hard and dense coatings are expected to have better mechanical and tribological properties than coatings with more delicate pores. In other words, it should not be forgotten that in cases where higher mechanical and tribological properties and lower corrosion sensitivity are desired, denser and thicker coatings are required [18,33].

It was seen that the Ca/P ratio of the coatings decreases with the decreasing pore size (Table 2 and Figure 5b,d). By the way, the Ca/P ratio of the coating reinforced with 1.0 wt% GNS is very close to the stoichiometric ratio of the free–GNS coating (include only nHA) (Figure 5a,c). It is likely that the increase of the ratio is due to the increasing pore diameters in the coatings. Larger pores may cause Ca, P, and O ions to come to the surface and be seen at higher rates. Therefore, the significant decrease of the Ca/P ratio in the 0.5 and 1.5 wt% GNS containing coatings may have been due to the formation of smaller pores in the coatings despite the increased number of micro-discharge channels and pores with the effect of GNS [18]. Some studies showed that the presence of graphene could close partial pores and reduce the diameter of some pores in the PEO coating [24,28]. The reduction of the pore diameter with the increasing of GNS led to the remaining Ca, P, and O ions in the coating and caused a reduced formation of pores in the layer. It was reported that the EDS analysis is a surface analysis, and Ca in particular is in the cathodic electron accumulation layer and also causes a decrease in the proportion of elements coming from substrates such as Ti, Al, or Nb [51]. The results showed that the GNS additive plays an active role in the elemental response of the coating, and its effect is mainly on the change of porosity content and the structure of the HA-based layer deposited on the surface. In particular, 1.0 wt% GNS doped HA coating has a high rate of Ca, P, and O elements, and the Ca/P ratio of the coating is significantly closer to the stoichiometric ratio of the HA (Figure 5c and Table 2). Besides, the lowest Ca/P ratio was seen in an HA composite coating with a 1.5 wt% GNS additive. As can be seen from Table 2, the Ca, P, and O elements in the coating were found in meager amounts. It has been reported in the literature that this is due to the higher amounts in the coating and lower pore size [24,28,51].

In this case, we expected to note a change in the Ca/P ratio. It could be concluded from XRD and EDS results that if the pores of the PEO treated coating have a bigger size, and lower number, the inner layer structures of the coating are transformed from amorphous to crystalline phases because of the high temperature and high pressure, and the diffusion of more Ca, P, and O ions into the coating structure [31,52]. Thus, there is no ideal Ca/P ratio. In other words, the ideal Ca/P ratio cannot be evaluated on its own. In short, it is well known that the Ca/P ratio of a structure can be changed according to formed phases in it, such as mono-, di- tri-, and tetra-calcium phosphate or hydroxyapatite [53]. Many studies showed that the Ca- and P- based apatite phases exist together in the same structure in many instances [5,54]. For example, BCP (biphasic calcium phosphate) includes both HA and β-TCP (tricalcium phosphate). It was reported that hybrid structures with a stable and coherent TiO2 layer increase the adhesion to the Ti substrates, mechanical resistance, and bioactivity of the coating while providing bioactivity inducing cell growth [18].

**Figure 6:**
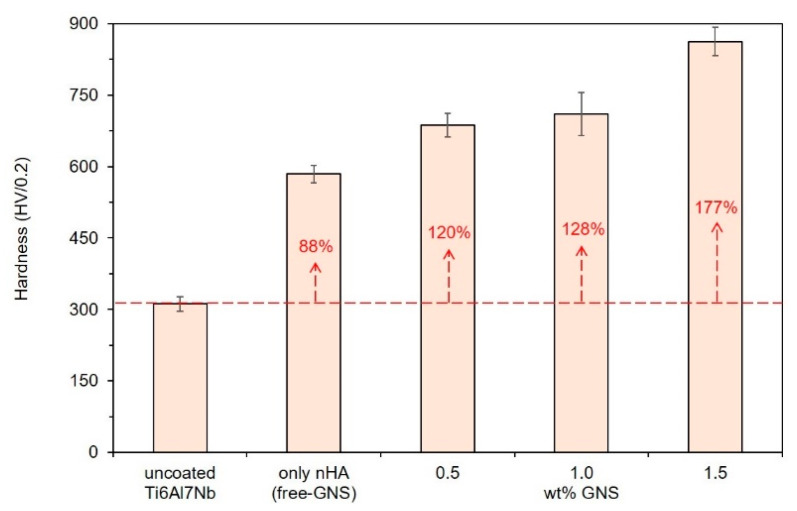
Microhardness results of the nHA/GNS hybrid coatings.

The measured microhardness values of the coatings reinforced with different GNS weight percentages, which were analyzed by using the ASTM E384 standard, are presented in Figure 6. The surface hardness values were increased by increasing the GNS quanta related to the transformation of anatase and rutile TiO_2_ phases. In other words, anatase and rutile TiO_2_ phases formed in the coating layer are the main reasons for the increase in hardness. Although GNS and HA are effective structures that are effective in these phases’ formation and transformation mechanisms, these structures have no direct effect on hardness measurements. It is well known that the potential phase, which provided low friction, high hardness, and wear-reductions, is the rutile TiO_2_ phase [55]. The Rutile TiO_2_ phase is thermodynamically more stable and harder than the anatase phase [56].

The rutile-based structures prevent the breaking of ceramic oxide coatings and increase the mechanical and abrasion resistance of the surface. The hardness of uncoated Ti6Al7Nb alloy was reported as 325 HV in the literature [57,58]. The hardness of the alloy was calculated as approximately 312 (±15) HV in the presented study. The XRD analyses were showed that the formation rate of the rutile TiO_2_ phase in the coating layer was increased with the increasing GNS quanta. In parallel, the highest surface hardness was obtained in the coating reinforced with 1.5 wt% GNS as about 862 (±30) HV. In other words, 1.5 wt% GNS addition to the coating provided an increase of about 48% and 177% in the coating hardness compared to free–GNS coating and uncoated structures, respectively. It could be foreseen that PEO treated coating presents higher resistance to abrasion and friction due to its high hardness values [52,57]. Generally, the thinner and nearer to hydroxyapatite crystalline coatings prefer mechanical strength and tribological performances [36]. However, the present study showed that the coating thickness, porosity content and size, GNS reinforcement ratio, and the type of Ca- and P-compounds and their ratio determine the biocompatibility of living cells and mechanical properties of nHA/GNS hybrid composite coatings. Thus, it could be concluded that the combination of these features is essential to obtain the best performance coatings.

**Figure 7:**
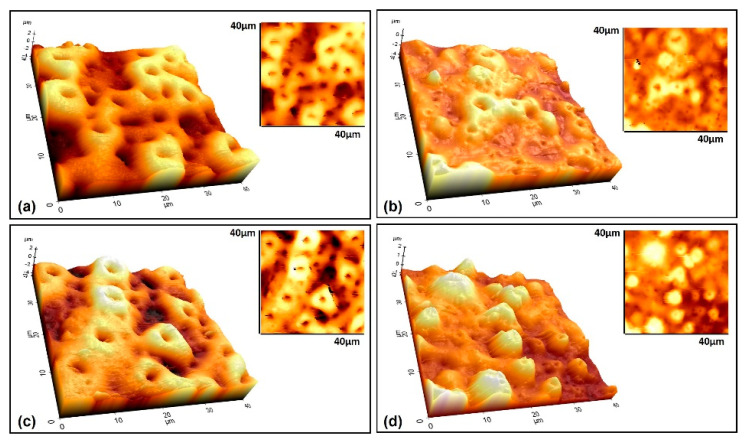
3D AFM topographic and wide–area (40 × 40 µm) surface images of the coatings reinforced with (**a**) free–GNS (only nHA), (**b**) 0.5, (**c**) 1.0, and (**d**) 1.5 GNS.

The 3D AFM images of the coating surfaces are given in Figure 7. In addition, the analysis results of the AFM characterizations were collected in Table 3.

By careful inspection of Figure 3 and Figure 7, it can be concluded that the GNS closes some pores during the PEO process, prevents the molten metal from ejected during the micro sparks, and thus, creates smaller but more pores. The surface areas of the coated Ti6Al7Nb alloys were calculated as 2141, 1815, 2378, and 1714 µm^2^ for free–GNS, 0.5, 1.0, and 1.5 wt% GNS containing coatings, respectively. Important distinctive parameters such as S_a_, S_q_, R_a_, and R_q_ increased in the beginning then decreased again with increasing GNS additive from 1.0 to 1.5 wt% (Table 3 and Figure 7). The lowest surface roughness values were obtained in 0.5 and 1.5 wt% GNS containing coatings. Similarly, the roughness values of the 1.0 wt% GNS and free–GNS coatings were very close to each other. The results agreed well with the pore size and numbers obtained from SEM observations (Table 1 and Figure 3). In other words, it could be said that the increase in the pore number of the coatings led to them being more homogeneous and having low roughness. Therefore, the most important factors of increasing the roughness and surface area of the coatings is the pore diameters and coating thickness indirectly.

**Figure 8:**
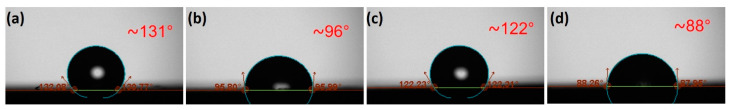
Contact angles of the coated surfaces (**a**) free–GNS and reinforced with (**b**) 0.5, (**c**) 1.0, and (**d**) 1.5 wt% GNS.

The surface wettability of coatings determines the ability of cells to attach to the surface. The contact angles of GNS doped and GNS-free coatings are shown with ±3° standard deviation values in Figure 8.

It is a well-known fact that there is an inverse correlation between the contact angle of surfaces and their wettability properties. Reducing the contact angle between the surfaces coated with SBF indicates increased wettability. The best hydrophilic property was obtained in the coating reinforced with 1.5 wt% GNS as 88°. Interestingly, the contact angles of the free–GNS and 1.0 wt% GNS containing coatings exhibited hydrophobic characteristics. These coatings are coatings with higher porosity and roughness than others. In these coatings, due to the large pore diameters, it was expected that the liquids would easily penetrate the pores and thus increased their wettability. Also, coatings with lower pore diameters and lower surface roughness showed better wettability than others. The most crucial factor in seeing this behavior may the higher number of pores in the coatings and the homogeneous distribution of these pores on the surface. As a result, the GNS additive changed the surface properties of the coatings and consequently significantly affected the wettability properties of the coatings.

**Figure 9:**
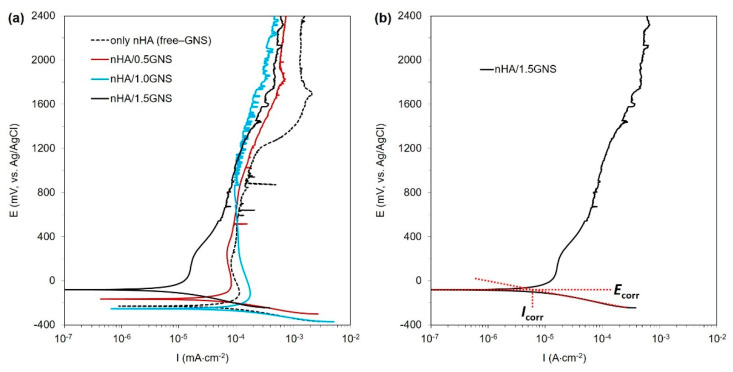
(**a**) The PDS graph of nHA/GNS hybrid coatings and (**b**) illustration of determining cathodic current density (*I*_corr_) from Tafel plot.

The PDS curves of the nHA/GNS coated samples were presented in Figure 9a. Besides, the corrosion potential (*E*_corr_), corrosion current density (*I*_corr_), corrosion rate, and polarization resistance (*R*_p_) values of the coatings calculated from the PDS curves are listed in Table 4. An illustration of determining current density (Icorr) from the Tafel plot is presented on 1.5 wt% GNS containing coatings in Figure 9b.

As is well known, the susceptibility of coatings to corrosion (thermodynamic aspect), the corrosion mechanism, and the corrosion rate (kinetics aspect) could be predicted by analyzing the PDS results. The *E*_corr_ is correlated to the thermodynamic aspect, describing the tendency of the sample to corrode, while *I*_corr_ is correlated to the kinetics aspect, which illustrates the corrosion rate of the tested sample. In general, a nobler *E*_corr_ and a lower *I*_corr_ usually indicate a lower corrosion rate and a better corrosion-protection property [16]. As shown in Table 4, the coating reinforced with 1.0 wt% GNS had the most negative *E*_corr_ (−253 mV) and the highest *I*_corr_ (75 µA cm^−2^), which suggested a higher corrosion susceptibility of this sample in comparison to other samples. This result was reflected by the largest value of corrosion rate and the lowest value of *R*_p_, as shown in Table 4. In contrast, the coating made with 1.5 wt% GNS showed the most noble values *E*_corr_ (−81 mV) and the lowest values of *I*_corr_ (7.0 µA cm^−2^) and *I*_cc_ (15 µA cm^−2^), implying that it has a higher corrosion resistance, as indicated by the lowest value of *I*_corr_ and the largest value of *R*_p_ in Table 4. This result indicated that the coating starts passivation earlier and is more resistant to corrosion. Such differences in the corrosion behavior of the samples would be related to the differences in the pore size of the coatings. The larger pores developed in the case of the free–GNS and 1.0 wt% GNS provided short paths for the corrosive species (SBF) to attack the substrate, which deteriorated the corrosion properties of these samples. On the other hand, the smaller and blocked pores developed in the coating 1.5 wt% GNS additive slow downed the motion of SBF towards the substrate. This meant that the addition of 1.5 wt% GNS to the coatings significantly reduced the coating’s corrosion rate, which is linked to denser structures containing closed and smaller pores [3,18,24].

**Figure 10:**
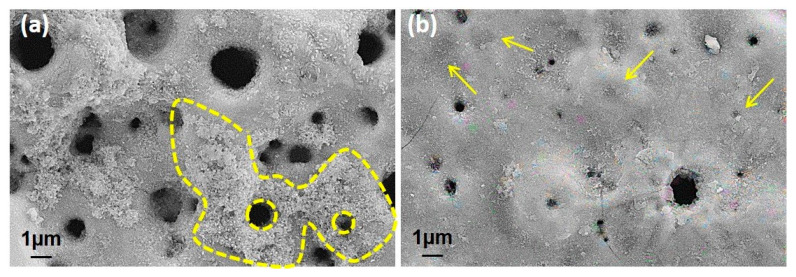
(**a**) The heavy corrosion tracks at the surroundings of pores in 1.0 wt% doped GNS coating and (**b**) closed pores due to the existence of GNS in the 1.5 wt% GNS coating.

**Figure 11:**
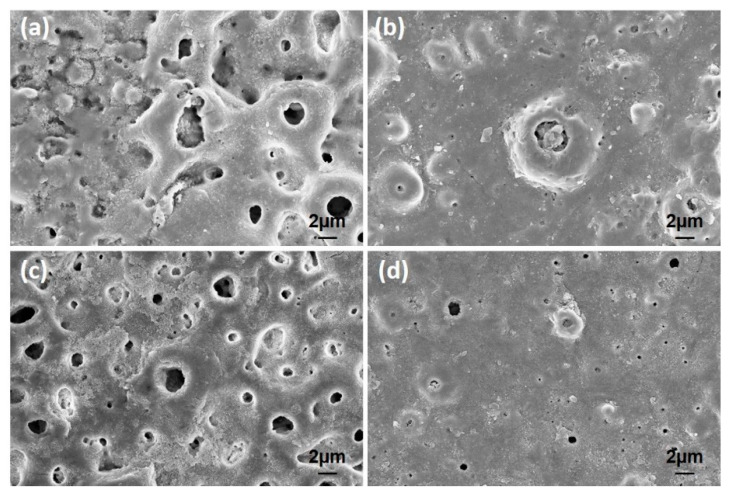
The surface SEM images of the coatings after corrosion tests in SBF: (**a**) free–GNS, (**b**) 0.5, (**c**) 1.0, and (**d**) 1.5 wt% GNS doped coatings.

The SBF in the smaller pores has a lesser wettability area than bigger pores and reduced the corrosive starting points. Thus, the low resistant areas formed in the pores and increased in H^+^ ion concentration in these pores. The process continued with dissolution on the surface and inside, which resulted in a big cathode/small anode corrosion due to the cathodic-based TiO_2_ layer on surfaces formed by PEO. As a result, the reduced pore sizes and higher coating thickness were essential factors, and they affected the corrosion resistance of the PEO treated coatings positively if the GNS acted as a barrier to the closing of pores [24,52,59]. In addition, Qaid et al. [35] and Ryu and Shrotriya [60] indicated that low roughness is one of the main factors that can affect the anti-corrosion properties of the surface. In this case, it could be considered that increasing pore sizes increases the roughness ratio on the surfaces and decreases the corrosion resistance in the 1.0 wt% containing coating. The heavy corrosion tracks at the environment of the pores and closed pores were clearly observed after corrosion tests on the 1.0 and 1.5 wt% GNS containing coatings, respectively (Figure 10). According to pore size in parallel, similar behavior was also observed in free–GNS and 0.5 wt% GNS doped coatings (Figure 11).

More corrosion damage was seen in free–GNS and 1.0 wt% GNS doped GNS coatings, as seen in Figure 11. The corrosion traces were only observed in large diameter pores in 0.5 and 1.5 wt% GNS doped coatings. By the way, the significant growth could be seen in the hexagonal rod-shaped crystalline form of HA after the corrosion tests, as compared with Figure 4 [61,62]. The HA clusters can be detected in many places on the surfaces. This proves that the HA structures are in nanocrystals form and that these crystals can be grown to become inorganic HA in SBF or within the human body on the PEO treated surfaces. In the literature, there are many studies about the growth of HA crystals on the surface after PEO (or hydrothermal treatment) in SBF [16,18,63,64,65].

**Figure 12:**
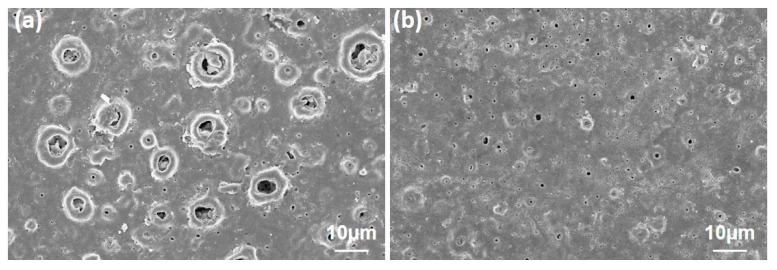
The corroded surfaces of (**a**) 0.5 and (**b**) 1.5 wt% GNS doped coatings at lower magnifications.

Corrosion caused damage not only in the pores but also in the hole areas remaining outside the large pores. However, when looking at lower magnifications, as can be seen in after corrosion SEM images, most of the GNS-coated samples and corrosion traces occurred in certain regions, while in some regions, the pores were also blocked (white rings in Figure 12). As has been already mentioned, the reason for this is that the graphene sheets cover specific areas of coated surface and prevent the corrosion of the liquid from leaking to the surface layers, thereby limiting the ion movement.

By the way, numerous forms of corrosion damage were reported on the PEO treated surfaces due to the microcracks caused by thermal shock [18,28,51,66,67]. Of course, surface cracks could also be seen in the coatings reinforced with GNS during the PEO. However, the GNS prevents forming cracks and proceeds on the surface.

## 3. Materials and Methods

### 3.1. Materials

Commercially Ti6Al7Nb alloy was supplied from FYTRONIX company (Elazig, Turkey) with dimensions of 500 × 500 × 4.1 mm. Graphene nanosheet (GNS) was purchased from Nanografi Company (Ankara, Turkey) with 99.9% pureness, 3 nm size, 800 m^2^ g^−1^ surface area, and 1.5 μm diameter. Calcium hydroxide (Ca(OH)_2_), sodium phosphate tribasic dodecahydrate (Na_3_PO_4_·12H_2_O), and sodium dodecyl sulfate (CH_3_(CH_2_)_11_OSO_3_Na) were used to prepare the electrolyte of PEO. The chemicals were provided by Sigma-Aldrich because of their analytically and high pureness and used without further purification. The substrates were cut from the Ti6Al7Nb sheets in the size of 25 × 25 × 4.1 mm. The chemical composition of the substrate is presented in Table 5. The substrates were ground with sandpapers and polished with 3 µm diamond paste to remove the oxide layer naturally formed on its surfaces and then ultrasonically cleaned with acetone and ethanol, respectively.

### 3.2. PEO Treatment

The electrolytes for PEO treatment were prepared usage with 2 g/L Ca(OH)_2_, 12 g/L Na_3_PO_4_·12H_2_O, and 0.5 mL sodium dodecyl sulfate (SDS) solution in distilled water. The GNS additive rates in the solutions were selected as 0.5, 1, and 1.5 wt%. The electrolytic bath was continuously mixed with a mixer to keep the GNS additive fully dispersed in a calcium and phosphate solution. The pH value and electrical conductivity were approximately measured as 12 and 12.3 mS cm^−1^ for the GNS-free electrolyte. The pH values of electrolytes including GNS, were showed an average of 12 but their electrical conductivities were altered and measured as 14.02, 15.02, and 15.06 mS cm^−1^ for 0.5, 1.0, and 1.5 wt% GNS doped solutions, respectively. The PEO working time was 10 min. and was performed with an AC power source by selecting bipolar PEO mode with a 10% duty cycle at 2000 Hz frequency. A steel tank with a 2 L volume was used as the cathode. The temperature was adjusted with a mixing and water cooling system in the range of 25–30 °C. All PEO operations were performed using 100 V negative and 500 V positive voltage.

### 3.3. In-Vitro Corrosion Tests

Ti-6Al-7Nb material was used as a substrate in this study, which can pass easily in body fluid. The corrosion monitoring of these type of materials requires wide scanning of potential ranges. Thus, the direct current potentiodynamic polarization scanning (DC PDS) technique was preferred to understand the corrosion kinetics of the coatings, as PDS tests can be used to measure localized corrosion and to determine whether or not a metal surface is passive. The alternate current electrochemical impedance spectroscopy (AC EIS) tests in which use low magnitude polarizing voltage, was not used in the presented study. The PDS tests were performed using a potentiostat/galvanostat (Gamry, PCI14/750, Warminster, PA, USA) equipment. An Ag/AgCl, Pt wire, and coated samples were used as a reference, auxiliary, and working electrodes during the tests, respectively. The tests were carried out in simulated body fluid (SBF) by using the potentiodynamic scanning (PDS) procedure. The SBF was also prepared according to Ref. [68]. The pH of the SBF was measured about 7.3 (±0.02) before the test, and its temperature was maintained at 37 (±0.5) °C to imitate human body conditions throughout the tests. Before starting PDS scanning, the samples were kept in the electrolyte to reach a steady-state open circuit potential (*E*_ocp_) for 40 min. After equilibrium, the polarization was started with a 1 mV·s^−1^ scan rate as dynamically from the potential at −0.3 V versus the *E*_ocp_ value, and when the anodic potential reached a potential of over 1.2 V, it was stopped. The exposed area of the samples was about 0.64 cm^2^, and all data were normalized according to the surface area. Then, the results were compared to the results of fee–GNS (only HA coated) samples under the same conditions. Corrosion susceptibilities of coated samples were evaluated by using EchemAnalyst software according to ASTM G102 [69]. All electrochemical tests were repeated at least three times and their reproducibility was in the gap of ±15 mV. The Gamry EchemAnalyst program also uses the Tafel approach based on the PDS experiments. To estimate *I*_corr_, a slope was drawn in the cathodic branch at +100 mV from the meeting point of anodic and cathodic curves (*E*_corr_) of the Tafel plot. The corrosion current of the point at which the slope line intersects with the horizontal line drawn from the *E*_corr_ was considered as Icorr, as shown in Figure 9b (marked with red dashed lines) [70,71]. By the way, although a 1 mV·s^−1^ was adopted for the experiments, for the potentiodynamic polarization curves, no substantial distortions were verified. Nevertheless, it is worth noting that the potential scan rate has an important role in order to minimize the effects of distortion in Tafel slopes and corrosion current density analyses, as previously reported [72,73,74,75].

### 3.4. Wettability Tests

The evolution of the wettability characteristics of the coated surfaces was compared with contact angle measurements. The tests were performed with the sessile drop technique (Biolin Scientific Attension^®^ Theta Flex, Manchester, UK) via usage SBF solution at body temperature. A digital camera recorded the shape of the SBF drop and the contact angles were recorded from the images digitally.

### 3.5. Hardness Measurements

Surface hardness was determined by using a micro Vickers hardness (Shimadzu, HMV-G, Columbia, MD, USA) technique under 1.961 N force with a loading time of 15 s according to the ASTM E384 standard [76]. The average hardness of the hybrid coatings was taken from randomly five different regions on the layers. A small force HV 0.2 (1.962 F) and a trace in a small area were measured in the microhardness test; no significant cracking or surface damage was observed during the test. Therefore, it was considered that the results describe the coatings in general. The microhardness values of the coatings exhibited a consistent behavior and no exaggerated finish was observed.

### 3.6. Characterization

The microstructural changes of the coatings before and after the corrosion tests were evaluated by a scanning electron microscope (SEM, Jeol, JSM-7001F, Tokyo, Japan). The compounds and products in the coating layers were characterized with energy-dispersive spectroscopy (EDS) attached to the SEM, X-ray diffraction (XRD, Bruker D8, Canton, MA, USA), and an attenuated total reflection infrared (ATR-FTIR, Thermo Scientific™ Nicolet™ iS™5, California, USA) spectroscopy. An atomic force microscope (AFM, Park System 100-E, Suwon, Korea) was used to evaluate the 3D surface structure of the coatings. The ImageJ program was used to measure the porosity data of coatings.

## 4. Conclusions

In the study, the nHA and GNS containing hybrid coatings from electrolytes containing calcium acetate and sodium phosphate and graphene were deposited on Ti6Al7Nb alloys successfully by the PEO method. The following conclusions can be drawn from the present study:

- The coatings had a porous structure due to the nature of the PEO process. The EDS and XRD results showed that the surfaces of the samples are covered with an oxide layer reinforced with Ca-/P-based and GNS containing hybrid structures.
- Anatase and rutile oxides, hydroxyapatite with very low intensities, and calcium pyrophosphate were mainly found in the coating layers. Besides, an nHA/GNS hybrid structure was confirmed by ATR-IR characterizations.
- The formation rate of the rutile TiO_2_ phase in the coating layer was increased with the increasing GNS addition. In parallel, the highest surface hardness was obtained in the coating reinforced with 1.5 wt% GNS as about 862 HV. In other words, 1.5 wt% GNS addition to the coating provided an increase of about 48% and 177% in the coating hardness compared to free–GNS coating and uncoated structures, respectively.
- It was observed that the pore diameters are significantly reduced in the coating reinforced with 0.5 wt% GNS additive. However, a small increase in the pore size was determined in the coating reinforced with 1.0 wt% GNS. Nevertheless, the pore diameters again decreased significantly with the addition of 1.5 wt% GNS. By the way, the number of pores increased with an increasing GNS addition, not due to their sizes.
- The results showed that the GNS additive plays an active role in the elemental response of the coating, and its effect is mainly on the change of porosity content and the structure of the HA-based layer deposited on the surface. In particular, 1.0 wt% GNS doped HA coating has a high rate of Ca, P, and O elements, and the Ca/P ratio of the coating is closer to the stoichiometric ratio of the HA. The lowest Ca/P ratio was seen in HA composite coating with a 0.5 wt% GNS additive. This is due to the higher amount of GNS in the coating and lower pore size.
- The lowest surface roughness values were obtained in 0.5 and 1.5 wt% GNS containing coatings. Similarly, the roughness values of the 1.0 wt% GNS and free–GNS coatings were very close to each other. The results were very much in agreement with the pore size and numbers obtained from SEM observations.
- The best hydrophilic property was obtained in the coating reinforced with 1.5 wt% GNS as 88°.
- The corrosion susceptibilities of the free–GNS and 1.0 GNS containing coatings were very similar to each other. On the other hand, the most resistant coating to corrosion was 1.5 wt% GNS containing coating. The Rp value of the sample was nine times higher compared to the one without a GNS sample. As a result, the reduced pore sizes and increasing coating thickness are important factors affecting corrosion resistance of the PEO treated coatings positively, if the GNS acts as a barrier to the closing of pores.

## Figures and Tables

**Table 1:** Coating thicknesses, porosity content, and surface areas of coated Ti6Al7Nb substrates with nHA/GNS provided by the PEO method.

Coating	Pore Number	Surf. Area (µm^2^)	Avg. Pore Dia. (µm)	Por. Ratio (%)	Coat. Thickness (µm)
only nHA	1804	2480	1.375	8.600	8.789
nHA/0.5GNS	3051	2102	0.689	3.862	7.617
nHA/1.0GNS	2560	4689	1.832	8.590	10.40
nHA/1.5GNS	2880	1955	0.679	3.592	4.164

**Table 2:** EDS analysis results of nHA/GNS coated Ti6Al7Nb alloys indicated in Figure 5 (at %).

Coating	Ti	Al	Nb	O	Ca	P	C
only HA	27.36	1.00	0.65	56.99	9.11	4.88	-
nHA/0.5 GNS	33.11	1.98	0.54	56.87	0.96	1.99	4.49
nHA/1.0 GNS	27.57	0.67	0.50	57.58	6.28	3.58	3.83
nHA/1.5 GNS	30.76	1.58	0.74	56.41	2.91	3.15	4.46

**Table 3:** The analysis results of the AFM characterizations performed on the nHA/GNS hybrid coatings.

Measurement Value	Only nHA (Free–GNS)	nHA/0.5 GNS	nHA/1.0 GNS	nHA/1.5 GNS
Min (μm)	−4.758	−4.758	−4.758	−1.280
Max (μm)	2.913	1.317	2.404	2.070
Mid (μm)	−0.923	−1.721	−1.177	0.395
Mean (μm)	0.495	−0.801	−0.312	−0.082
R_pv_ (μm)	7.671	6.076	7.162	3.350
R_q_ (μm)	1.130	0.588	0.974	0.566
R_a_ (μm)	0.946	0.461	0.769	0.442
R_z_ (μm)	7.612	5.931	6.961	3.165
R_sk_	0.322	−0.753	0.428	−0.703
R_ku_	2.571	3.851	3.930	3.175
S_a_ (µm)	1.032	0.8885	0.7894	0.4587
S_q_ (µm)	1.237	0.9942	1.022	0.572
Area (µm^2^)	2141	1815	2378	1714

**Table 4:** Corrosion parameters calculated from PDS curves of the coatings.

Coating	*E*_corr_ (mV)	*I*_cor_ (×10^−6^, A·cm^−2^)	*I*_cc_ (×10^−6^, A∙cm^−2^)	Corr. Rate (×10^−6^, mm·yr^−1^)	*R*_p_ (ohms·cm^2^)
only nHA (free–GNS)	−229	60	115	0.944	450,006
nHA/0.5GNS	−165	47	83	0.739	608,018
nHA/1.0GNS	−253	75	178	1.180	218,178
nHA/1.5GNS	−81	7	15	0.110	2,724,289

**Table 5:** Chemical composition of Ti6Al7Nb substrates.

Composition	Al	Nb	Fe	N	O	C	Ti
Ti6Al7Nb	6.12	7.07	0.12	0.01	0.18	0.02	Bal.

## Data Availability

The data presented in this study are available on request from the corresponding author. We confirm, if any data needed we can provide it.

## Sample Availability

Samples are available from the authors.
